# *Astragalus* polysaccharides exerts immunomodulatory effects *via* TLR4-mediated MyD88-dependent signaling pathway *in vitro* and *in vivo*

**DOI:** 10.1038/srep44822

**Published:** 2017-03-17

**Authors:** Lijing Zhou, Zijing Liu, Zhixue Wang, Shuang Yu, Tingting Long, Xing Zhou, Yixi Bao

**Affiliations:** 1 Department of Clinical Laboratory, the Second Affiliated Hospital of Chongqing Medical University, Chongqing, China; 2 Department of Clinical Medicine, Xinjiang Medical University, Xinjiang, China

## Abstract

*Astragalus* polysaccharides (APS), which is widely used as a remedy to promote immunity of breast cancer patients, can enhance immune responses and exert anti-tumor effects. In this study, we investigated the effects and mechanisms of APS on macrophage RAW 264.7 and EAC tumor-bearing mice. Griess reaction and ELISA assays revealed that the concentrations of nitric oxide, TNF-α, IL-1β and IL-6 were increased by APS. However, this effect was diminished in the presence of TAK-242 (TLR4 inhibitor) or ST-2825(MyD88 inhibitor). In C57BL/10J (TLR4^+/+^wild-type) and C57BL/6J (MyD88^+/+^wild-type) tumor-bearing mice, the tumor apoptosis rate, immune organ indexes and the levels of TNF-α, IL-1β and IL-6 in blood increased and the tumor weight decreased by oral administration of APS for 25 days. APS had no obvious effects on IL-12p70. However, these effects were not significant in C57BL/10ScNJ (*TLR4*-deficient) and C57BL/B6.129P2(SJL)-*Myd88*^*m1.1Defr*^/J (*MyD88*-deficient) tumor-bearing mice. qRT-PCR and Western blot indicated that APS stimulated the key nodes in the TLR4-MyD88 dependent signaling pathway, including TLR4, MyD88, TRAF-6, NF-κB and AP-1, both *in vitro* and *in vivo*. However, TRAM was an exception. Moreover, TRAF-6 and NF-κB were not triggered by APS in *gene*-deficient tumor-bearing mice. Therefore, APS may modulate immunity of host organism through activation of TLR4-mediated MyD88-dependent signaling pathway.

Breast cancer is the most common cancer among women and the leading cause of cancer-related death worldwide^1^. Traditional therapies, including surgical resection, radiotherapy and chemotherapy, are predominately applied to prolong survival time of cancer patients. However, chemoradiotherapy could cause severe side-effects in advanced cancer. At present, traditional Chinese medicine (TCM) has been well accepted as a complementary and alternative therapy to improve the clinical symptoms, control the tumor size, improve the quality of life and prolong the survival time for cancer patients^2^,^3^,^4^.

Various kinds of polysaccharides from TCM show anti-tumor activities and immunoregulation effects^5^,^6^,^7^,^8^. *Astragalus* polysaccharides (APS), the main active extract from *Astragalus membranaceus*, has been well recognized as an anti-tumor immunomodulator and widely applied in clinic^9^,^10^. It has a molecular weight of 3.6 × 10^4^ Da and has a variety of bio-activities^11^,^12^,^13^,^14^,^15^. The immunomodulatory^16^ and anti-tumor activities^17^,^18^ are its main functions.

The toll-like receptors (TLRs) play important roles in identifying pathogens and activating the innate immune system^19^,^20^. TLR4 is the first identified Toll protein on cell membranes, which can recognize lipopolysaccharides (LPS) of Gram-negative bacteria^21^. Besides, TLR4 on macrophages is essentially involved in many natural polysaccharide-induced events^5^. It also plays a crucial role in the enhancement of innate immune response and the production of cytokines induced by polysaccharides^22^. The branches of TLR4 signaling pathways include TLR4-toll interleukin1 receptor adaptor protein (TIRAP)/MAL-myeloid differentiation factor 88 (MyD88) (MyD88-dependent) and TRAM/Toll/IL-1 receptor (TIR)-domain-containing adaptor-inducing interferon (TRIF) (MyD88-independent) signaling pathways. MyD88-dependent signaling pathway is involved in several immunological disorders^23^,^24^. A study was designed to identify and characterized the receptors of APS on immune cells. The results demonstrated that there was direct interaction between APS and TLR4 on cell surface of macrophages^25^. It was also reported that polysaccharides of *Radix Astraguli* induced cytokine production in RAW264.7 cells through TLR4-mediated activation of MAPKs and NF-κB^18^. However, whether the immunomodulatory and anti-tumor effects of APS is mediated through TLR4 signaling pathway is still unclear.

Here, we sought to systematically identify and characterize the immunoregulation and anti-tumor effects of APS *in vitro* and *in vivo*. Moreover, this study was also designed to investigate the role and mechanism of TLR4 signaling pathway in APS-induced immunoregulation and tumor inhibition.

## Materials and Methods

### Reagents

APS, which was dissolved in aseptic phosphate buffer saline (PBS), was purchased from Kamai Shu Biotechnology (Shanghai, China) with a purity of 98.5% for *in vitro* experiments. The possibility of LPS contamination in the APS was ruled out by chromogenic end-point tachypleus amebocyte lysate (CE TAL) assay kit (Chinese Horseshoe Crab Reagent Manufactory Co. Ltd., Xiamen, China). The APS powder was from Xi’an yuensun biological technology company (Xi’an, Shaanxi province, China) with a purity of 70% and was dissolved in normal saline at 65 °C for oral administration. LPS was isolated from *Escherichia coli* 055:B5, which was bought from Biosharp (Anhui, China). Adriamycin (ADM) was gained from Shenzhen Main Luck Pharmaceuticals Inc. (Shenzhen, China). The ADM powder was dissolved in normal saline injections according to the manufacturer’s instructions. Dulbecco’s modified Eagle’s medium (DMEM), trypsin and dialyzed fetal bovine serum (FBS) were obtained from Hyclone (Logan city, UT, USA). TAK-242 (Resatorvid), a small-molecule specific inhibitor of Toll-like receptor 4 (TLR4), and ST-2825, a MyD88 pharmacologic inhibitor, were purchased from MedChem Express (MCE) (NJ, USA). The ELISA kits for cytokine detection were acquired from 4A biotech Co. Ltd. (Beijing, China). The Universal RNA Extraction Kit, PrimeScript RT reagent Kit with gDNA Eraser and SYBR *Premix Ex Taq*^TM^ II were gained from TaKaRa Bio Inc. (Kyoto, Japan). Immunoblotting antibodies were purchased from Cell Signaling Technology (Beverly, MA). Annexin-V-FITC apoptosis detection kit and Collagenase IV were from Sigma-Aldrich Co. Ltd. (St. Louis, MO, USA).

### Animals

Female, 4–6 weeks old, weighing 18–22 g, C57BL/10ScNJ mice (TLR4-deficient mice lacking functional *tlr4*, TLR4^−/−^) and C57BL/B6.129P2 (SJL)-*Myd88*^*m1.1Defr*^/J mice (MyD88-deficient mice lacking functional *myd88,* MyD88^−/−^), as well as their corresponding control group C57BL/10J mice (wild-type mice, TLR4^+/+^) and C57BL/6J mice (wild-type mice, MyD88^+/+^) were purchased from Model Animal Research Center of Nanjing University (Nanjing, China). EAC cells obtained from Nanjing KeyGEN biotech Co. Ltd. (Nanjing, China) were diluted to a concentration of 1 × 10^7^ cells/ml with sterilized physiological saline and inoculated into the right armpit of each mouse. The solid-tumor-bearing mice model was established as described previously^22^. All the tumor-bearing mice of each murine strain were randomly distributed into four groups (n = 8 each): normal saline (NS) group (orally administered with the same volume of normal saline as the APS group once a day for 25 days), ADM group (4 mg/kg/d, intraperitoneal injection for the first 3 days followed with the same treatment as NS group), APS group (500 mg/kg/d, orally administered for 25 days), LPS group (5 mg/kg, intraperitoneal injection 4 hours before sacrifice after orally administered with NS once a day for 25 days). After 25 days, the eyeball blood was collected. The weight of tumor, spleen and thymus were measured. The organ indexes of spleen and thymus and tumor inhibition rate were calculated according to the following formulas: organ indexes (%) = mean weight of organ/body weight × 100%; inhibition rate (%) = (1-mean weight of tumor in the administration groups/mean weight of tumor in the control group) × 100%.

All the animal experiments were approved and performed in accordance with the guidelines of Institutional Animal Care and Use Committee of Chongqing Medical University. Mice were maintained at Experimental Animal Center of Chongqing Medical University under pathogen-free conditions with free access to food and water. All possible steps were taken to avoid animals’ suffering at each stage of the experiments with the use of appropriate and adequate anesthesia.

### Cell culture

The murine macrophage-like cell line RAW 264.7 was gifted by Professor Jianping Gong from Department of Hepatobiliary Surgery, the Second Affiliated Hospital of Chongqing Medical University. The cells were cultured in DMEM supplemented with 10% (v/v) FBS and antibiotics (100 U/ml penicillin and 100 μg/ml streptomycin) at 37 °C in a humid atmosphere with 5% CO_2_. RAW 264.7 cells were seeded in culture plates and treated with or without APS (400 μg/ml) or LPS (100 ng/ml). Then, the cell culture supernatants were collected. In the experiments with inhibitors, the cells were pre-cultured for 3 h with 20 μg/ml TAK-242 or ST2825 prior to incubation with 400 μg/ml APS or 100 ng/ml LPS for 24 h.

### Griess reaction

RAW 264.7 cells were seeded (5 × 10^4^ cells/ml) in 24-well culture plates and treated with or without APS (400 μg/ml) or LPS (100 ng/ml) for different periods of time (4 h, 8 h, 16 h, 24 h, 32 h, 48 h and 72 h) *in vitro*. The volume of cell culture medium was 0.5 ml. The culture supernatants in different time groups were collected at each time point for the analysis. NO_2_^−^ accumulation was used as an indicator of nitric oxide (NO) production. And, the nitrite content in the culture supernatant was determined by Griess reaction as previously described^7^.

### Measurement of apoptosis by flow cytometry

Tumor tissues was minced into small pieces and then digested by collagenase type IV with a concentration of 10 mg/ml for 2 h at room temperature. After digestion, the cells were washed twice in DMEM and then washed in PBS. Tumor cell apoptosis was detected by flow cytometry using Annexin-V-FITC apoptosis detection kit according to the instructions.

### Enzyme-linked Immunosorbent assay (ELISA)

RAW 264.7 cells were seeded (5 × 10^5^ cells/ml) in 24-well culture plates and treated with or without APS (400 μg/ml) or LPS (100 ng/ml) for 24 h. The levels of TNF-α, IL-6, IL-1β and IL-12p70 were detected in serum and culture supernatant collected at 24 h of incubation. ELISA was performed using a commercial ELISA kit (4A biotech Co. Ltd., China) according to the manufacturer’s instructions.

### Quantitative real-time PCR assay (qRT-PCR)

The total RNA of spleen homogenates was extracted and reverse transcribed into cDNA. qRT-PCR was performed on Bio-Rad CFX-96 (Bio-Rad, Foster City, CA, USA). The condition of qRT-PCR amplification was as follows: initial denaturation for 30 s at 95 °C, followed by 40 cycles of denaturation at 95 °C for 5 s, annealing and extension at 56 °C for 30 s. Reduced glyceraldehyde-phosphate dehydrogenase (GAPDH) and β-actin served as internal controls. The relative mRNA expressions of TLR4, MyD88, TRAM, TRAF-6, NF-κB and AP-1 were calculated by Vandesompele Method^26^. The sequences of the primers were listed in Table 1. Triplicate reactions were run per sample and each experiment was repeated three times.

### Western blot

The proteins were extracted from the spleen tissues and the treated RAW 264.7 macrophage cells. Then, proteins were separated by 10% sodium dodecyl sulfate-polyacrylamide gel and electro-blotted onto polyvinylidene difluoride membranes (Immunobilon TM-P; Millipore, USA). After blocking with 5% BSA in TBST buffer (Tris 10 mM, NaCl 150 mM, pH 7.6, 0.1% Tween 20), the membranes were probed with primary and secondary antibodies. Protein bands were visualized by enhanced chemiluminescence (Millipore) and analyzed with ChemiDoc Imaging system (Bio-Red, USA).

### Statistical analysis

SPSS 17.0 software (SPSS Inc., Chicago, IL, USA) was used for statistical analysis. Data were shown as mean ± standard deviation (SD). Differences between two groups were assessed by unpaired two-tailed Student’s *t*-test. Data sets that involved more than two groups were assessed by one-way analysis of variance (ANOVA). Differences were considered statistically significant at *P* < 0.05.

## Results

### *In vitro* Experiments

#### APS increases the secretion of immunomodulatory factors by RAW 264.7 macrophages

**Figure 1:**
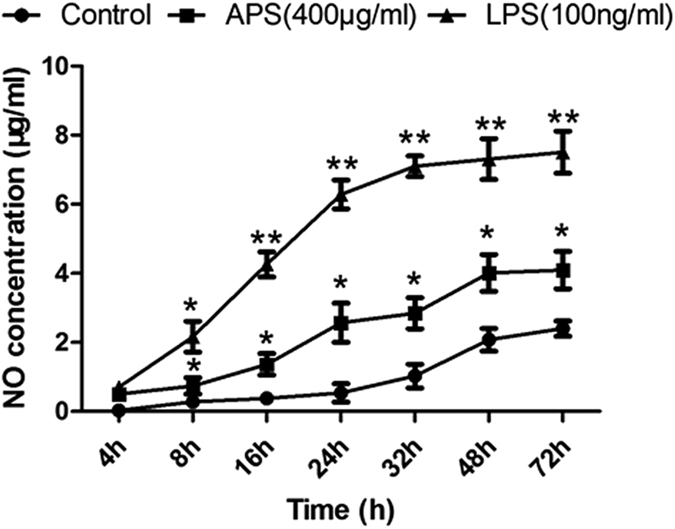
Effects of APS on NO production by RAW 264.7 macrophage *in vitro*. Macrophages were cultured with APS (400 μg/ml) or LPS (100 ng/ml) for 4–72 h. The supernatants were assayed for NO production by Griess reaction. *P < 0.05, **P < 0.01 *vs*. Control group.

To investigate the immunomodulatory effects of APS on macrophages, the levels of NO and cytokines in the culture supernatants of RAW 264.7 cells were detected by Griess reaction and ELISA. A preliminary test determined that the optimal dose and optimal incubation time of APS were 400 μg/ml and 24 h for RAW 264.7 cells *In vitro*. As shown in Fig. 1, the NO production was significantly increased by APS and LPS, remained relative high level from 8 h to 72 h (*P* < 0.05). As shown in Table 2, the treatment of APS significantly increased the IL-1β, IL-6 and TNF-α secretion compared with control group (*P* < 0.05). However, APS had no significant effect on IL-12p70 (*P* > 0.05). The results suggest that APS can stimulate RAW 264.7 macrophages to secrete NO, IL-1β, IL-6 and TNF-α *in vitro*.

#### APS has effects on the expressions of mRNAs and proteins of TLR4 signaling pathway in RAW 264.7 macrophages

**Figure 2:**
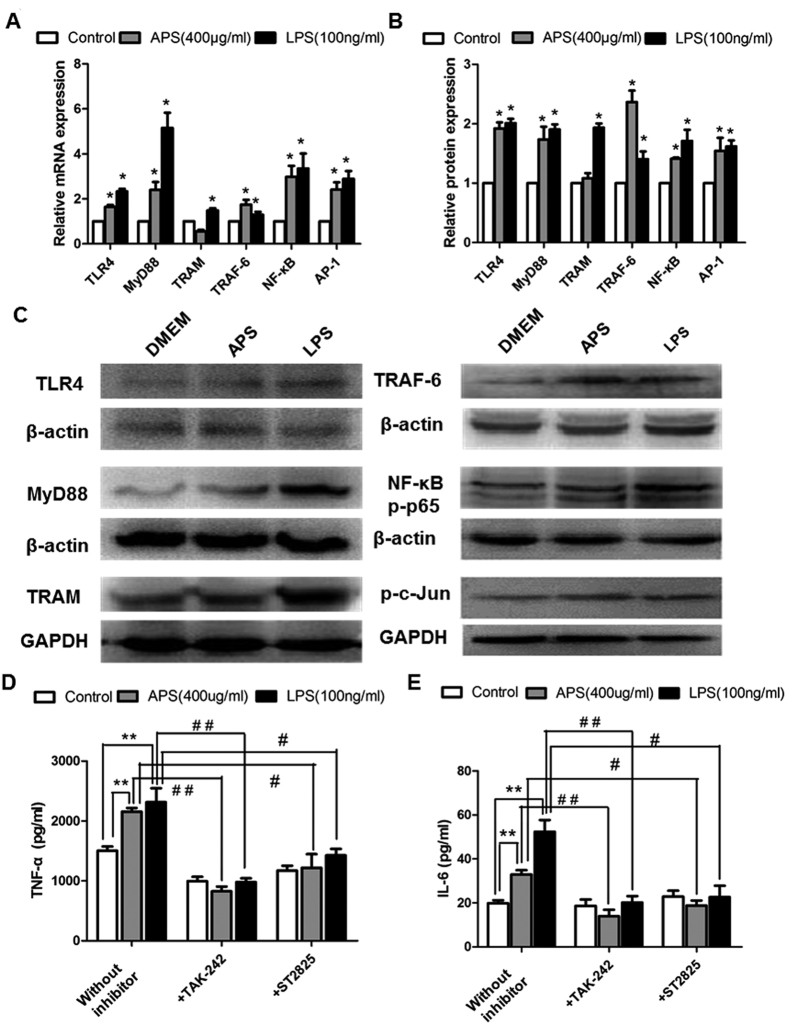
Effects of APS on TLR4 signaling pathway in RAW 264.7 cells. The RAW 264.7 cells were incubated with APS (400 μg/ml) or LPS (100 ng/ml) for 24 h. (**A**) The correlative genes expression of the key nodes in TLR4 signaling pathway were detected by qRT-PCR. (**B**) Quantification of Western blots for the key-node proteins as fold-changes relative to β-actin or GAPDH. (**C**) The bands of Western blot. Each column represents the mean ± SD of at least three independent experiments. *P < 0.05 vs. Control group. RAW 264.7 macrophages were pre-incubated with or without inhibitors of TLR4 (TAK-242) and MyD88 (ST-2825) for 3 h and then treated with APS (400 μg/ml) or LPS (100 ng/ml) for 24 h. The secretion of TNF-α (**D**) and IL-6 (**E**) were analyzed by ELISA. The reported values are the mean ± SD, n = 3. *P < 0.05, **P < 0.01. ^#^*P* < 0.05, ^##^*P* < 0.01.

To explore the immunoregulatory mechanism of APS, the expressions of mRNAs and proteins of the key nodes (TLR4, MyD88, TRAM, TRAF-6, NF-κB and AP-1) in TLR4 signaling pathway were detected using qRT-PCR (Fig. 2A) and Western blot (Fig. 2B and C). After incubation with APS or LPS for 24 h, the mRNA and protein expression levels of TLR4, MyD88, TRAF-6, NF-κB and AP-1 were significantly elevated compared with those in the control group (all *P* < 0.05). In contrast, the mRNA and protein expression levels of TRAM in the APS group were not significantly different from those in the control group (*P* > 0.05). These results suggest that APS activates TLR4 signaling pathway but selectively up-regulates the mRNA and protein expressions of some key nodes. These results also imply that the effects of immunoregulation by APS are probably mediated through TLR4 signaling pathway.

#### APS promotes immunomodulatory effects of RAW 264.7 macrophages via TLR4 and MyD88

To further analyze whether TLR4 and MyD88 are involved in APS-induced macrophage activation, cells were pre-treated with TAK-242 and ST2825, which are inhibitors of TLR4 and MyD88, respectively. Then, ELISA was performed to detect cytokine levels of TNF-α and IL-6. As shown in Fig. 2D and E, in cells without inhibitors, the production of TNF-α and IL-6 was significantly increased by APS and LPS, compared with those in control group (*P* < 0.05). However, with the presence of inhibitors, the TNF-α and IL-6 production induced by APS were suppressed and were significantly lower than those without inhibitors (*P* < 0.05). The results confirm that the effect of APS on immunoregulation in macrophages is probably acted through TLR4 and MyD88.

### *In vivo* Experiments

#### APS inhibits tumor weight and facilitates immune organ indexes and cytokines secretion in EAC-bearing mice

**Figure 3:**
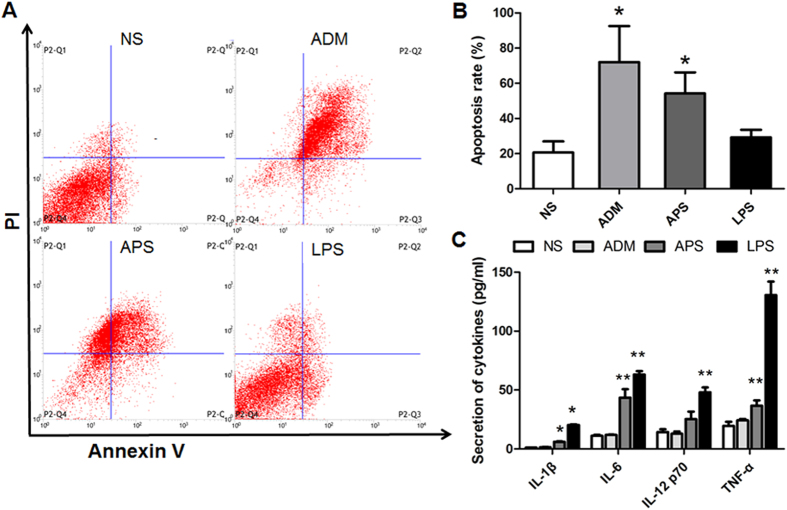
Effects of APS on tumor inhibition and immunoregulation *in vivo*. (**A**) The tumor cell apoptosis of EAC tumor-bearing mice detected by flow cytometry. (**B**) The comparison of tumor cell apoptosis rate in each group. (**C**) The secretion of cytokines in serum of EAC tumor-bearing mice. *P < 0.05, **P < 0.01 vs. Control group.

To further investigate the immunoregulatory effect of APS *in vivo*, EAC tumor-bearing mice were used. EAC cells were diluted and inoculated into the right armpit of each mouse. The solid-tumor-bearing mice model was established and treated as described in the Materials and Methods section. Adriamycin (ADM), commonly used in the treatment of cancers^27^, was used as the positive control for the *in vivo* experiments. Tumor weight was analyzed to evaluate tumor inhibition by APS. As shown in Table 3, the tumor weight in APS group and ADM group was significantly declined, compared with those in the NS group (*P* < 0.05). Moreover, the tumor cells apoptosis of EAC tumor-bearing mice were detected by flow cytometry. As shown in Fig. 3A,B, cell apoptosis rate in NS group, ADM group, APS group and LPS group was 20.65 ± 12.56, 72.04 ± 29.03, 54.22 ± 23.93 and 29.21 ± 6.07, respectively. Compared with NS group, the apoptosis rate in APS group and ADM group were significantly increased (*P* < 0.05), while those in LPS group had no obvious changes (*P* > 0.05).

For immunomodulation, the weight of thymus and spleen was measured and organ index was calculated (Table 2). The thymus and spleen index of APS group were significantly higher than those in NS group (*P* < 0.05). However, the thymus and spleen index had no significant differences between in ADM group and NS group (*P* > 0.05). Furthermore, cytokines in serum of EAC tumor-bearing mice were analyzed by ELISA. As shown in Fig. 3C, the levels of IL-1β, IL-6 and TNF-α were significantly elevated by APS and LPS, compared with those in NS group (*P* < 0.05). In LPS group, IL-12p70 level was also significantly increased than NS group. However, APS had no significant influence on the level of IL-12p70 (*P* > 0.05). ADM has no significant effects on the levels of all detected cytokines in mice peripheral blood (*P* > 0.05). The results indicate that APS has significant immunoregulatory and anti-tumor effects on tumor-bearing mice.

#### Anti-tumor immunomodulatory effects of APS are probably mediated by TLR4 signaling pathway

To identify whether TLR4 signaling pathway participates in the anti-tumor and immunomodulatory effects of APS *in vivo*, C57BL/10 J (TLR4^+/+^) and TLR4-deficient (TLR4^−/−^) tumor-bearing mice were applied in this study. The expression of the key nodes in the TLR4 signaling pathway was detected by qRT-PCR and Western blot. The cytokine levels in serum were analyzed by ELISA.

**Figure 4:**
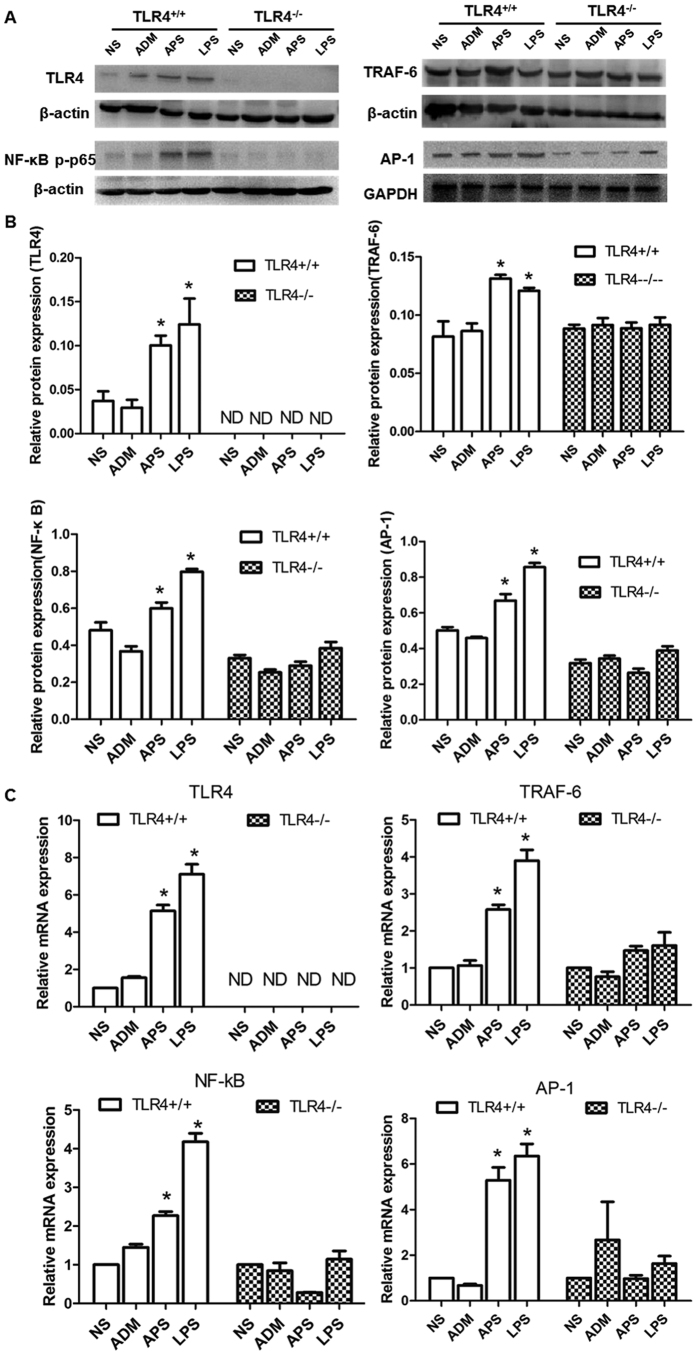
Effects of APS on mRNA and proteins expression of TLR4 signaling pathway in tumor-bearing mice. The mRNA and protein levels of TLR4, TRAF-6, NF-κB and AP-1 in different groups of the EAC tumor-bearing mice with TLR4^+/+^/TLR4^−/−^ were detected by qRT-PCR and Western blot, respectively. (**A**) Representative Western blot results of TLR4, TRAF-6, NF-κB and AP-1 expression. (**B**) Relative protein expression of TLR4, TRAF-6, NF-κB and AP-1. (**C**) Relative mRNA expression of TLR4, TRAF-6, NF-κB and AP-1. Each column represents the mean ± SD of at least three independent experiments. *P< 0.05, as compared with the corresponding control group without APS treatment. ND = not detected.

As shown in Fig. 4, APS and LPS were found to significantly induce the expressions of mRNAs and proteins of TLR4, TRAF-6, NF-κB and AP-1 in the TLR4^+/+^ tumor-bearing mice, compared with those in the NS group (*P* < 0.05). Inversely, there was no remarkable differences in the expression of mRNA and proteins of TLR4, TRAF-6, NF-κB and AP-1 among groups in the TLR4^−/−^ tumor-bearing mice (*P* > 0.05).

**Figure 5:**
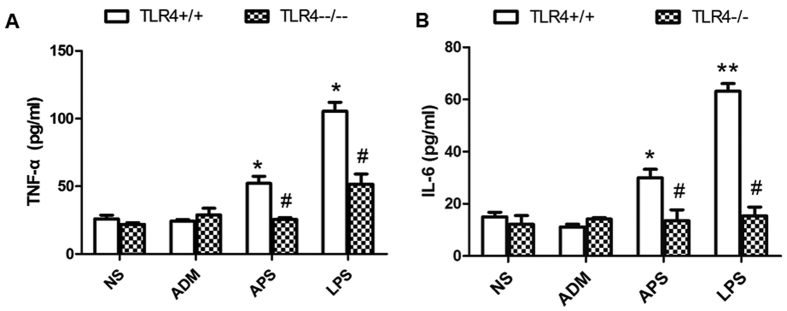
Effects of APS on the cytokine production in serum of TLR4^+/+^/TLR4^−/−^ tumor-bearing mice. The cytokines in the blood of tumor-bearing mice were analyzed by ELISA. The production of TNF-α (**A**) and IL-6 (**B**) in different groups of TLR4^+/+^/TLR4^−/−^ tumor-bearing mice. *P< 0.05, as compared with the corresponding control group without APS treatment. ^#^*P*< 0.05, as compared with the corresponding groups in their wild-type tumor-bearing mice.

In TLR4^+/+^ tumor-bearing mice, the levels of TNF-α and IL-6 (Fig. 5A,B) in APS and LPS groups were significantly higher than those in NS and ADM TLR4^+/+^ groups (*P* < 0.05). In TLR4^−/−^ tumor-bearing mice, the concentrations of TNF-α and IL-6 had no significant differences among groups (*P* > 0.05). Additionally, the secretion of cytokines in the TLR4^−/−^ mice of the APS and LPS groups were significantly lower than those in the TLR4^+/+^ mice (*P* < 0.05).

In summary, APS induces the expressions of mRNAs and proteins of TLR4, TRAF-6, NF-κB and AP-1, as well as increasing the levels of TNF-α and IL-6 in the serum of the TLR4^+/+^ tumor-bearing mice, but not TLR4^−/−^ tumor-bearing mice. This indicates that TLR4 signaling pathway is probably involved in the anti-tumor and immunomodulation effects induced by APS.

#### Anti-tumor immunomodulatory effects of APS are mediated by TLR4-MyD88-dependent signaling pathway

To clarify the detailed mechanism of anti-tumor immunomodulatory effects of APS and to verify whether APS activates TLR4 signaling pathway through MyD88-dependent pathway, we used C57BL/6J (MyD88^+/+^) and MyD88-deficient (MyD88^−/−^) tumor-bearing mice in this study.

**Figure 6:**
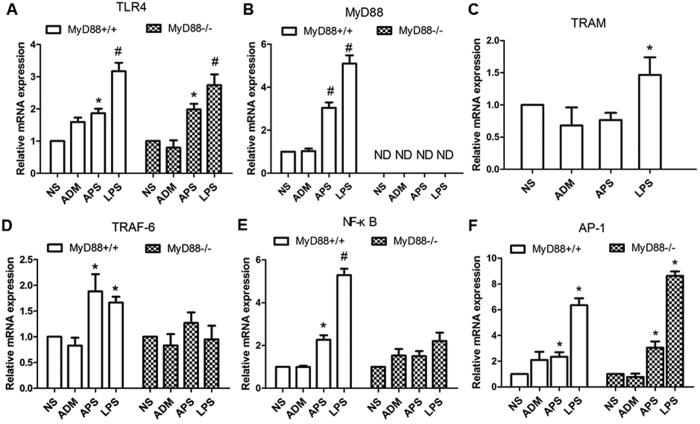
Effects of APS on mRNA expressions of TLR signaling pathways in MyD88^+/+^/MyD88^−/−^ tumor-bearing mice. The relative mRNA expressions of TLR4 (**A**), MyD88 (**B**), TRAF-6 (**D**), NF-κB (**E**) and AP-1 (**F**) in different groups of MyD88^+/+^/MyD88^−/−^ EAC tumor-bearing mice were detected by qRT-PCR. And the mRNA expression of TRAM (**C**) was only detected in the groups of the MyD88^+/+^ mice. Each column represents the mean ± SD of at least three independent experiments. *P< 0.05, as compared with the corresponding control group without APS treatment. ND = not detected.

**Figure 7:**
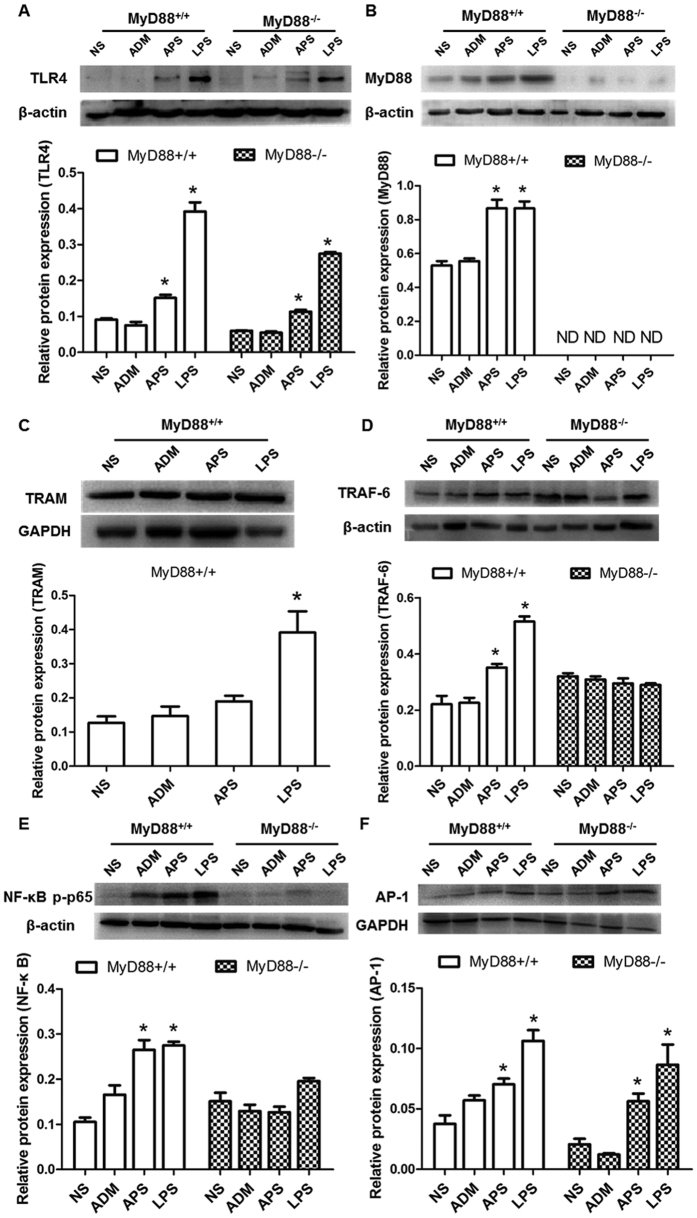
Effects of APS on protein expressions of TLR signaling pathways in MyD88^+/+^/MyD88^−/−^ tumor-bearing mice. The protein expressions of TLR4 (**A**), MyD88 (**B**), TRAF-6 (**D**), NF-κB (**E**) and AP-1 (**F**) in different groups of the EAC tumor-bearing mice with MyD88^+/+^/MyD88^−/−^ were detected by Western blot. And the protein and mRNA expression of TRAM (**C**) was only detected in the groups of the MyD88^+/+^ mice. Each column represents the mean ± SD of at least three independent experiments. *P< 0.05, as compared with the corresponding control group without APS treatment. ND = not detected.

The mRNA and protein expressions of TLR4, MyD88, TRAF-6, NF-κB and AP-1 were detected by qRT-PCR and Western blot (Figs 6 and 7). Results showed that the mRNA and protein levels were obviously provoked by APS and LPS in the MyD88^+/+^ tumor-bearing mice, compared with those in the NS group (*P* < 0.05). However, there was no significant difference in the mRNA and protein expressions of TRAF-6 and NF-κB among groups in the MyD88^−/−^ tumor-bearing mice (*P* > 0.05). TLR4 is in the up-stream of the pathway. Therefore, the deficiency of MyD88 slightly affected the induction of TLR4 expression by APS and LPS (Figs 6 and 7). On the contrary, the mRNA and protein expressions of TRAM had no remarkable differences among the groups of the MyD88^+/+^ tumor-bearing mice. The mRNA and protein expressions of AP-1 were increased by APS and LPS in both MyD88^+/+^ mice and MyD88^−/−^ mice.

**Figure 8:**
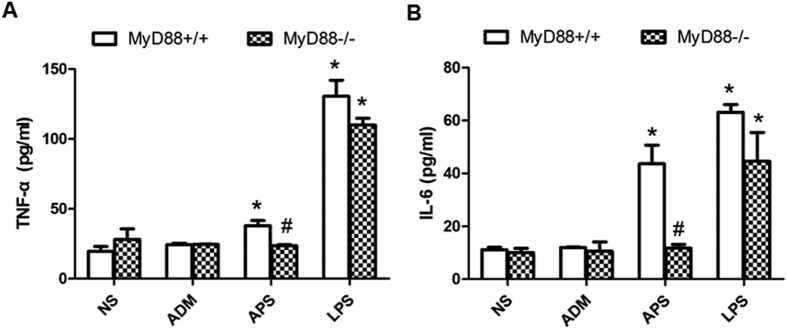
Effects of APS on the cytokine production in serum of MyD88^+/+^/MyD88^−/−^ tumor-bearing mice. The cytokines in the blood of tumor-bearing mice were analyzed by ELISA. The concentrations of TNF-α (**A**) and IL-6 (**B**) in different groups of MyD88^+/+^/MyD88^−/−^ tumor-bearing mice. Each column represents the mean ± SD of at least three independent experiments. *P< 0.05, as compared with the corresponding control group without APS treatment. ^#^*P*< 0.05, as compared with the corresponding groups in their wild-type tumor-bearing mice.

Moreover, both TNF-α and IL-6 in the serum of the C57BL/6J (MyD88^+/+^) tumor-bearing mice stimulated by APS and LPS were obviously higher than those in NS and ADM groups (*P*< 0.05, Fig. 8A and B). The concentrations of TNF-α and IL-6 in the APS group had no obvious difference from those in the NS group of the MyD88^−/−^ tumor-bearing mice (*P* > 0.05). In addition, the secretion of cytokines after treatment with APS in the MyD88^−/−^ mice were significantly reduced, compared with those in the APS groups of the MyD88^+/+^ tumor-bearing mice (*P* < 0.05). The positive control LPS also significantly increased TNF-α and IL-6 levels in both MyD88+/+ and MyD88−/− mice (P < 0.05). However, the levels of TNF-α and IL-6 in LPS groups had no significant differences between in MyD88+/+ and MyD88−/− mice (P > 0.05). All these results suggest that APS can selectively activate the down-stream key nodes and the terminal effect factors of TLR4-MyD88-dependent pathway *in vivo*.

## Discussion

APS, have been shown to have multiple immunomodulatory functions, such as inhibiting the proliferation of CD4^+^CD25^+^ regulatory T cells^10^, promoting the maturation of dendritic cells (Shao *et al*., 2016), regulating the imbalance of Thl/Th2 subgroups, regulating the differentiation of the erythroid lineage^28^,^29^, and enhancing the cytostatic activity of macrophages^29^. Macrophages are specific antigen-presenting cells that play important roles in anti-infection and anti-tumor immunity through engulfing and eradicating pathogens^7^. There are two approaches for activated macrophages to immunomodulate and kill tumor cells or pathogens, including direct contact or releasing cytotoxic molecules such as NO and cytokines^30^.

NO, synthesized by macrophage-induced nitric oxide synthase, has been identified as a major effect molecule involved in many biological processes^31^, including pathogen elimination^32^ and destruction of tumor cells by activated macrophages^33^. IL-1β, IL-6, IL-12p70 and TNF-α are typical multifunctional cytokines involved immune responses, hematopoiesis and inflammation. IL-1β has a wide range of immunomodulaory effects and may mediated inflammation or be directly involved in the inflammatory process^34^. IL-6 is a cytokines with multiple immunomodulatory functions, and it can stimulate B cells, T cells and stem cell proliferation, promote the B cell production of immnoglobulin and promote cytotoxic lymphocyte and stem cell differentiation^35^ IL-12p70, the active heterodime, is known as a T-cell stimulating factor which can stimulate the growth and function of T cells^36^. TNF-α generated by macrophages is implicated in cytotoxic function in certain tumors^5^, and in the development and procession of immunoregulatroy effects of APS^37^,^18^. Our result showed that APS could directly increase the NO, IL-1β, IL-6 and TNF-α production by macrophages *in vitro,* but not IL-12p70. Similarly, the level of IL-1β, IL-6 and TNF-α was also increased by APS in EAC tumor-bearing mice *in vivo*. APS was reported to improve the spleen/thymus indexes of H22 tumor bearing mice^38^. In this study, the immune organ indexes of tumor-bearing mice were also higher than those in NS group. These data indicate that APS improves the secretion of immunomodulatory factors *in vitro* and *in vivo.*

As mentioned before, APS is wildly accepted as a complementary and alternative therapy for cancer patients^9^,^10^. APS is also reported to inhibit the growth of breast cancer cell line MDA-MB-468^17^ and enhance the therapeutic effect of cisplatin^39^. It is found that the anti-tumor effect of APS on H22 tumor-bearing mice might be related to its ability to enhance the expression of IL-1, IL-2, IL-6 and TNF-α and decrease of IL-10^40^. In the present work, in tumor bearing mice, the tumor weight was inhibited by APS and the tumor cell apoptosis rate was increased by APS. Together, these results suggest that APS has anti-tumor activity in tumor-bearing mice. And, this effect may be acted *via* regulating the production of cytokines.

Subsequently, we explored the underlying mechanism of APS. TLR4 signaling pathways have two branches: MyD88-dependent and MyD88-independent signaling pathways^19^ MyD88, containing a death domain in cytoplasm^11^, is bound to the structural domain of TLR by MyD88 adaptor-like (MAL) protein, which is an essential adapter protein binding to MyD88 and activating the downstream molecule TRAF-6^39^. TRAM, with a similar role as MAL, is able to combine with TRIF and TLR^41^. and is specifically involved in the MyD88-independent signaling pathway^19^. It delivers TRIF to the endosomes via a specific region of the plasma membrane^41^. TRAF-6, is also recruited to the complex of TRAM/TRIF to regulate the expression of correlated cytokines and interferon type I/II^42^. Therefore, TRAF-6 can be considered as the intersection of the two types of TLR4 signaling pathway, which can activate NF-κB and MAPK^7^ to regulate the expression of the downstream key nodes^43^. In addition, AP-1 is the heterodimer of c-Fos and c-Jun^44^, whose binding sites are regulated by TNF-α through MAPKs-mediated pathway^45^. In view of these, we detected the mRNA and protein levels of TLR4, TRAF-6, NF-κB and AP-1 in RAW 264.7 cells. Results showed that the expressions of the key nodes were significantly induced by APS, compared with the control group *in vitro*. Besides, the mRNA and protein expressions of TLR4, TRAF-6, NF-κB and AP-1 in the splenocytes of the TLR4^+/+^ EAC tumor-bearing mice were also observed increased significantly. Furthermore, the APS-induced TLR4, TRAF-6, NF-κB and AP-1 expression were decreased in the splenocytes of the TLR4^−/−^ EAC tumor-bearing mice. TAK-242 (Resatorvid), a small-molecule specific inhibitor of TLR4, binds selectively to TLR4 and interferes with the interactions between TLR4 and its adaptor molecules^46^,^47^. The APS had slight influence on the secretions of TNF-α and IL-6 in RAW 264.7 macrophages treated with TLR4 inhibitors (TAK-242). The data indicates that TLR4 involves in APS-mediated immune activation. This result partly supports the opinion of the study implying directly interaction between APS and TLR4 on cell surface^25^. Moreover, APS-induced immunoregulation may *via* TLR4 signaling pathway.

**Figure 9:**
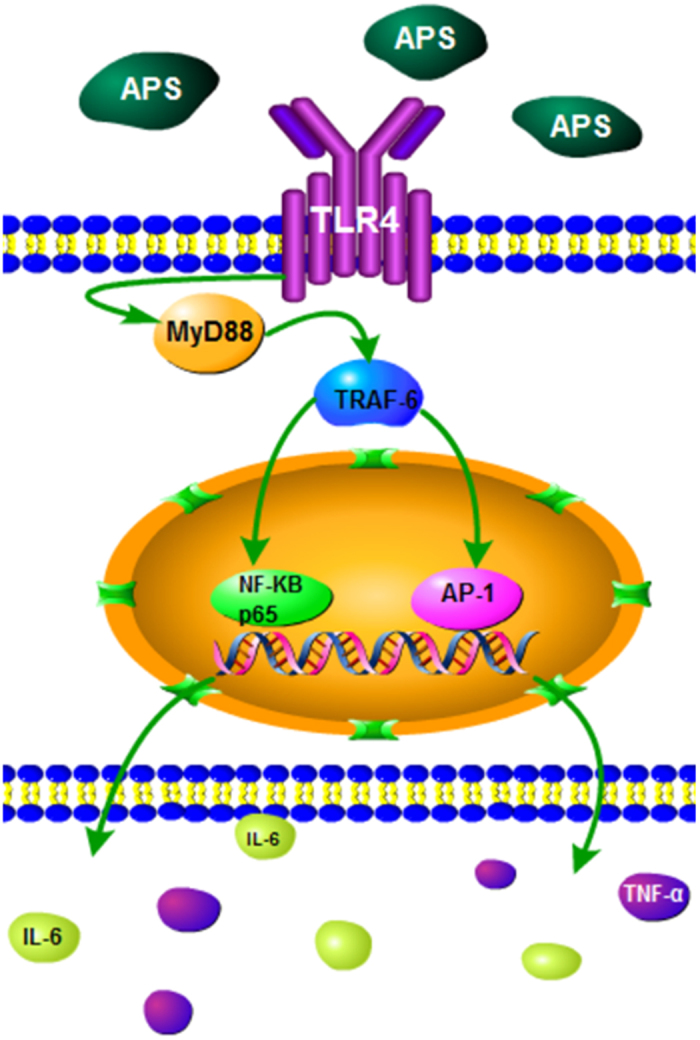
The proposed mechanism underlying the immunoregulation and anti-tumor effects of APS. The TLR4-mediated MyD88-dependent signaling pathway is probably one of mechanisms underlying the immunoregulation and anti-tumor effects of APS.

To give a good insight into the deep mechanism of APS immunoregulation and to deeply verified which branch of TLR4 signaling pathway is involved in APS-mediated immune activation, the key nodes expression in RAW 264.7 cells, MyD88^+/+^ and MyD88^−/−^ tumor-bearing mice were detected. TRAM, specifically involved in the MyD88-independent signaling pathway, had no obvious changes with treatment of APS both in RAW 264.7 cells and in MyD88^+/+^ tumor-bearing mice. The mRNA and protein expressions of TRAF-6, NF-κB and AP-1 in the splenocytes of MyD88^−/−^ tumor-bearing mice were not provoked by APS. And the improvement of APS on the secretion of IL-6 and TNF-α was also suppressed by ST-2825. ST-2825, a MyD88 pharmacologic inhibitor, is widely used to impede the dimerization of MyD88^48^. The activation of TLR4 signaling pathway by APS were almost lost when TLR4 and MyD88 were deficient or inhibited. Taken together, we suppose that APS may activate the TLR4-MyD88 dependent pathway through TLR4 (Fig. 9). Then, the key nodes in the TLR4-MyD88 dependent pathway, including TRAF-6, NF-κB and AP-1, are activated. Finally, the production of effector cytokines such as IL-6 and TNF-α is enhanced to mediate the immunomodulation effects of APS.

However, exceptions still exist. It is confusing that the mRNA and protein expressions of AP-1 were increased by APS and LPS in both MyD88^+/+^ mice and MyD88^−/−^ mice. AP-1, which is another eukaryotic transcription factor targeted by MAPK signaling pathways^49^, is an important regulatory protein involved in various biological activities, and also contributes to inflammatory and immune responses^50^. Thus, we assumed that, when MyD88 was deficient, APS activated AP-1 through another signaling pathway other than the TLR4-MyD88-denpendent signaling pathway. On account of the complexities of signal transduction with various intersections, we speculated that APS might provoke other signaling pathways through TRAM or might have potential relationship with AP-1 when *MyD88* was deficient. However, no relative reports are found. The detailed mechanism of this phenomenon needs to be thoroughly investigated in future studies.

In conclusion, the TLR4-mediated MyD88-dependent signaling pathway is probably one of the APS-induced signal pathways underlying the immunoregulation and anti-tumor effects of APS both *in vitro* and *in vivo*.

## Additional Information

**How to cite this article:** Zhou, L. *et al. Astragalus* polysaccharides exerts immunomodulatory effects *via* TLR4-mediated MyD88-dependent signaling pathway *in vitro* and *in vivo. Sci. Rep.* **7**, 44822; doi: 10.1038/srep44822 (2017).

**Publisher's note:** Springer Nature remains neutral with regard to jurisdictional claims in published maps and institutional affiliations.

## Figures and Tables

**Table 1:** The sequences of primers used in this study.

TLR4	forward	5′-CTGGGTGAGAAAGCTGGTAA-3′
	reverse	5′-AGCCTTCCTGGATGATGTTGG-3′
MyD88	forward	5′-GTTGTGTGTGTCCGACCGT-3′
	reverse	5′-GTCAGAAACAACCACCACCATGC-3′
TRAM	forward	5′-GGCCTGGACCATCTTGTTAC-3′
	reverse	5′- CATGGGTATGACGGAGTTGT-3′
TRAF-6	forward	5′-CATCTTCAGTTACCGACAGCTCAG-3′
	reverse	5′-TGGTCGAGAATTGTAAGGCGTAT-3′;
NF-κB	forward	5′-CCAAAGAAGGACACGACAGAATC-3′
	reverse	5′-GGCAGGCTATTGCTCATCACA-3′
AP-1	forward	5′-GGCAGGCTATTGCTCATCACA-3′
	reverse	5′-GAAGTTGCTGAGGTTGGCCTA-3′
β-Actin	forward	5′-AGCTTACTGCTCTGGCTCCTAGC-3′
	reverse	5′-ACTCATCGTACTCCTGCTTGCT-3′
GAPDH	forward	5′-GACATCAAGAAGGTGGTGAAGC-3′
	reverse	5′-GAAGGTGGAAGAGTGGGAGTT-3′.

**Table 2:** The level of cytokines in culture supernatant of RAW264.7 cells (mean ± SD, n = 5).

Groups	IL-1β (pg/ml)	IL-6 (pg/ml)	IL-12 p70 (pg/ml)	TNF-α (pg/ml)
Control	3.812 ± 2.97	19.92 ± 2.30	43.02 ± 1.46	1503.47 ± 120.02
APS (400 μg/ml)	8.83 ± 2.24*	32.96 ± 3.39**	39.93 ± 2.08	2156.06 ± 105.43**
LPS (100 ng/ml)	9.63 ± 2.06*	52.34 ± 9.23**	84.23 ± 10.31*	2312.97 ± 402.06**

Note: *P < 0.05 vs Control group; **P < 0.01 vs Control group.

**Table 3:** The comparison of tumor weight and immune organ indexes of EAC tumor-bearing mice (mean ± SD, n ≥ 4).

Groups	Tumor weight (g)	Thymus index (%)	Spleen index (%)
NS	2.27 ± 0.58	0.060 ± 0.024	0.59 ± 0.12
ADM (4 mg/kg/d)	0.61 ± 0.24**	0.048 ± 0.0084	0.38 ± 0.14
APS (500 mg/kg/d)	1.39 ± 0.53*	0.13 ± 0.042*	1.07 ± 0.36*

Note: *P < 0.05 vs NS group. **P < 0.01 vs NS group.

## References

[b1] Ferlay J. . Estimates of worldwide burden of cancer in 2008: GLOBOCAN. Int. J. Cancer 127, 2893–2917 (2010). 21351269 10.1002/ijc.25516

[b2] Tian Q. E. . Astragalus polysaccharides can regulate cytokine and P-glycoprotein expression in H22 tumor-bearing mice. World. J. Gastroenterol. 18, 7079–7086 (2012). 23323011 10.3748/wjg.v18.i47.7079 PMC3531697

[b3] Guo H. R., Liu J. X., Xu L., Madebo T. & Baak J. P. Traditional Chinese Medicine Herbal Treatment May Have a Relevant Impact on the Prognosis of Patients with Stage IV Adenocarcinoma of the Lung Treated With Platinum-Based Chemotherapy or Combined Targeted Therapy and Chemotherapy. Integr. Cancer Ther. 10, 127–137 (2011). 21147812 10.1177/1534735410387599

[b4] Jiang Y. . Traditional Chinese Medicine treatment as maintenance therapy in advanced non-small-cell lung cancer: A randomized controlled trial. Complement. Ther. Med. 24, 55–62 (2016). 26860802 10.1016/j.ctim.2015.12.006

[b5] Hsu H. Y. . Extract of Reishi Polysaccharides Induces Cytokine Expression via TLR4-Modulated Protein Kinase Signaling Pathways. J. Immunol. 173, 5989–5999 (2004). 15528333 10.4049/jimmunol.173.10.5989

[b6] Zhu Z. Y. . Effects of extraction methods on the yield, chemical structure and anti-tumor activity of polysaccharides from Cordyceps gunnii mycelia. Carbohydr. Polym. 140, 461–471 (2016). 26876874 10.1016/j.carbpol.2015.12.053

[b7] Feng Z., Wang Z., Yang M., Zhou L. & Bao Y. Polysaccharopeptide exerts immunoregulatory effects via MyD88-dependent signaling pathway. Int. J. Biol. Macromol. 82, 201–207 (2016). 26546866 10.1016/j.ijbiomac.2015.11.002

[b8] Cheng Y. . The effects of polysaccharides from the root of Angelica sinensis on tumor growth and iron metabolism in H22-bearing mice. Food Funct. 7, 1033–1039 (2016). 26757699 10.1039/c5fo00855g

[b9] Guo L., Bai S. P., Zhao L. & Wang X. H. Astragalus polysaccharide injection integrated with vinorelbine and cisplatin for patients with advanced non-small cell lung cancer: effects on quality of life and survival. Med. Oncol. 29, 1656–1662 (2012). 21928106 10.1007/s12032-011-0068-9

[b10] Li Q., Bao J. M., Li X. L., Zhang T. & Shen X. H. Inhibiting effect of Astragalus polysaccharides on the functions of CD4 + CD25 high Treg cells in the tumor microenvironment of human hepatocellular carcinoma. Chin. Med. J. 125, 786–793 (2012). 22490576

[b11] Jiang J., Wu C., Gao H., Song J. & Li H. Effects of Astragalus polysaccharides on immunologic function of erythrocyte in chickens infected with infectious bursa disease virus. Vaccine 28, 5614–5616 (2010). 20598783 10.1016/j.vaccine.2010.06.025

[b12] Jiang J. B. . Therapeutic effects of astragalus polysaccharides on inflammation and synovial apoptosis in rats with adjuvant-induced arthritis. Int. J. Rheum. Dis. 13, 396–405 (2010). 21199477 10.1111/j.1756-185X.2010.01555.x

[b13] Li R., Chen W. C., Wang W. P., Tian W. Y. & Zhang X. G. Antioxidant activity of Astragalus polysaccharides and antitumour activity of the polysaccharides and siRNA. Carbohydr. Polym. 82, 240–244 (2010).

[b14] Zhao L. H., Ma Z. X., Zhu J., Yu X. H. & Weng D. P. Characterization of polysaccharide from Astragalus radix as the macrophage stimulator. Cell Immunol 271, 329–334 (2011). 21937031 10.1016/j.cellimm.2011.07.011

[b15] Jia R., Cao L., Xu P., Jeney G. & Yin G. *In vitro* and *in vivo* hepatoprotective and antioxidant effects of Astragalus polysaccharides against carbon tetrachloride-induced hepatocyte damage in common carp (Cyprinus carpio). Fish Physiol. Biochem. 38, 871–881 (2012). 22089693 10.1007/s10695-011-9575-z

[b16] Jin M., Zhao K., Huang Q. & Shang P. Structural features and biological activities of the polysaccharides from Astragalus membranaceus. Int. J. Biol. Macromol. 64, 257–266 (2014). 24325861 10.1016/j.ijbiomac.2013.12.002

[b17] Ye M. N., Chen H. F., Zhou R. J. & Liao M. J. Effects of Astragalus polysaccharide on proliferation and Akt phosphorylation of the basal-like breast cancer cell line. Zhong Xi Yi Jie He Xue Bao 9, 1339–1346 (2011). 22152773 10.3736/jcim20111210

[b18] Wei W. . TLR-4 may mediate signaling pathways of Astragalus polysaccharide (RAP) induced cytokine expression of RAW264.7 cells. J. Ethnopharmacol. 179, 243–252 (2016). 26743224 10.1016/j.jep.2015.12.060

[b19] Yamamoto M. . TRAM is specifically involved in the Toll-like receptor 4–mediated MyD88-independent signaling pathway. Nat. Immunol. 4, 1144–1150 (2003). 14556004 10.1038/ni986

[b20] Chen X. . Sargassum fusiforme polysaccharide activates nuclear factor kappa-B (NF-κB) and induces cytokine production via Toll-like receptors. Carbohydr. Polym. 105, 113–120 (2014). 24708959 10.1016/j.carbpol.2014.01.056

[b21] Mai C. W. . Mechanisms Underlying the Anti-Inflammatory Effects of Clinacanthus nutans Lindau Extracts: Inhibition of Cytokine Production and Toll-Like Receptor-4 Activation. Front. Pharmacol. 7, 7 (2006). 10.3389/fphar.2016.00007 PMC4735445 26869924

[b22] Wang Z., Dong B., Feng Z., Yu S. & Bao Y. A study on immunomodulatory mechanism of Polysaccharopeptide mediated by TLR4 signaling pathway. BMC Immunology 16, 34–43 (2015). 26032186 10.1186/s12865-015-0100-5 PMC4450994

[b23] Li W. . Immunomodulatory effects of polysaccharopeptide (PSP) in human PBMC through regulation of TRAF6/TLR immunosignal-transduction pathways. Immunopharmacol. Immunotoxicol. 32, 576–584 (2010). 20131955 10.3109/08923971003586876

[b24] Verstak B. . The TLR signaling adaptor TRAM interacts with TRAF6 to mediate activation of the inflammatory response by TLR4. J. Leukoc. Biol. 9, 427–436 (2014). 10.1189/jlb.2A0913-487R PMC4632169 24812060

[b25] Shao B. M. . A study on the immune receptors for polysaccharides from the roots of Astragalus membranaceus, a Chinese medicinal herb. Biochem. Biophys. Res. Commun. 320, 1103–1111 (2004). 10.1016/j.bbrc.2004.06.065 15249203

[b26] Hellemans J., Mortier G., De Paepe A., Speleman F. & Vandesomepele J. qBase relative quantification framework and software for management and automated analysis of real-time quantitative PCR data. Genome. Biol. 8, R19 (2007). 17291332 10.1186/gb-2007-8-2-r19 PMC1852402

[b27] Tacar O., Sriamornsak P. & Dass C. R. Doxorubicin: an update on anticancer molecular action, toxicity and novel drug delivery systems. The Journal of Pharmacy and Pharmacology 65, 157–170 (2013). 23278683 10.1111/j.2042-7158.2012.01567.x

[b28] Li R. J., Qiu S. D., Chen H. X., Tian H. &Wang H. X. The immunotherapeutic effects of Astragalus polysaccharide in type 1 diabetic mice. Biol. Pharm Bull. 30, 470–476 (2007). 17329840 10.1248/bpb.30.470

[b29] Yang M., Qian X. H., Zhao D. H. & Fu S. Z. Effects of astragalus polysaccharide on the erythroid lineage and microarray analysis in K562 cells. J. Ethnopharmacol. 127, 242–250 (2010). 19922785 10.1016/j.jep.2009.11.013

[b30] Cho W. C. & Leung K. N. *In vitro* and *in vivo* anti-tumor effects of Astragalus membranaceus. Cancer Lett. 252, 43–54 (2007). 17223259 10.1016/j.canlet.2006.12.001

[b31] Ando I. . Safflower polysaccharides activate the transcription factor NF-κB via Toll-like receptor 4 and induce cytokine production by macrophages. Int. Immunopharmacol. 2, 1155–1162 (2002). 12349952 10.1016/s1567-5769(02)00076-0

[b32] Niedbala W., Ca B. & Liew F. Y. Role of nitric oxide in the regulation of T cell functions. Ann. Rheum. Dis. 65, iii37–40 (2006). 17038470 10.1136/ard.2006.058446 PMC1798386

[b33] Hou Y. C., Janczuk A. & Wang P. G. Current trends in the development of nitric oxide donors. Curr. Pharm. Des. 5, 417–441 (1999). 10390607

[b34] Faggioni R., Fantuzzi G., Fuller J., Dinarello C. A. & Feingold K. R. IL-1β mediates leptin induction during inflammation. Am. J. Physiol. 274, 204–208 (1998). 10.1152/ajpregu.1998.274.1.R204 9458919

[b35] Akira S., Hirano T., Taga T. & Kishimoto T. Biology of multifunctional cytokines, IL-6 and related molecules (IL-1 and TNF). FASEB J. 4, 2860–2867 (1999). 2199284

[b36] Wang K. S., Frank D. A. & Ritz J. Interleukin-2 enhances the response of natural killer cells to interleukin-12 through up-regulation of the interleukin-12 receptor and STAT4. Blood 95, 3183–3190 (2000). 10807786

[b37] Luan A. . Astragalus polysaccharide attenuates isoproterenol-induced cardiac hypertrophy by regulating TNF-α/PGC-1α signaling mediated energy biosynthesis. Environ. Toxicol. Pharmacol. 39, 1081–1090 (2015). 10.1016/j.etap.2015.03.014 25880160

[b38] Yang B., Xiao B. & Sun T. Antitumor and immunomodulatory activity of Astragalus membranaceus polysaccharides in H22 tumor-bearing mice. Int. J. Biol. Macromol. 62, 287–290 (2013). 24060282 10.1016/j.ijbiomac.2013.09.016

[b39] Ming H. . Astragalus polysaccharides combined with cisplatin decreases the serum levels of CD44 and collagen type IV and hyaluronic acid in mice bearing Lewis lung cancer. Xi Bao Yu Fen Zi Mian Yi Xue Za Zhi 31, 909–913 (2015). 26146060

[b40] Tian Q. E. . Effects of Astragalus polysaccharides on P-glycoprotein efflux pump function and protein expression in H22 hepatoma cells *in vitro*. BMC. Complement. Altern. Med. 12, 94 (2012). 22784390 10.1186/1472-6882-12-94 PMC3493361

[b41] Kim S. J. . Suppression of TRIF-dependent signaling pathway of toll-like receptors by allyl isothiocyanate in RAW 264.7 macrophages. Int. Immunopharmacol. 13, 403–407 (2012). 22668719 10.1016/j.intimp.2012.05.017

[b42] Rowe D. C. . The myristoylation of TRIF-related adaptor molecule is essential for Toll-like receptor 4 signal transduction. Proc. Natl. Acad. Sci. USA 103, 6299–6304 (2006). 16603631 10.1073/pnas.0510041103 PMC1458872

[b43] Luo T. . Astragalus polysaccharide attenuates lipopolysaccharide-induced inflammatory responses in microglial cells: regulation of protein kinase B and nuclear factor-κB signaling. Inflamm. Res. 64, 205–212 (2015). 25669325 10.1007/s00011-015-0798-9

[b44] Hsieh H. L., Lin C. C., Shih R. H., Hsiao L. D. & Yang C. M. NADPH oxidase-mediated redox signal contributes to lipoteichoic acid-induced MMP-9 upregulation in brain astrocytes. J. Neuroinflamm. 9, 110 (2012). 10.1186/1742-2094-9-110 PMC3391180 22643046

[b45] Saito Y. . Disruption of group IVA cytosolic phospholipase A(2) attenuates myocardial ischemia-reperfusion injury partly through inhibition of TNF-alpha-mediated pathway. Am. J. Physiol. Heart. Circ. Physiol. 302, 2018–2030 (2012). 10.1152/ajpheart.00955.2011 PMC3362116 22427514

[b46] Naoko M., Noboru T., Tatsumi M. & Masayuki I. TAK-242 (Resatorvid), a Small-Molecule Inhibitor of Toll-Like Receptor (TLR) 4 Signaling, Binds Selectively to TLR4 and Interferes with Interactions between TLR4 and Its Adaptor Molecules. Mol. Pharmacol. 79, 34–41 (2011). 20881006 10.1124/mol.110.068064

[b47] Salama M. . Toll-like receptor 4 blocker as potential therapy for acetaminophen-induced organ failure in mice. Exp. Ther. Med. 10, 241–246 (2015). 26170942 10.3892/etm.2015.2442 PMC4487059

[b48] Hernanz R. . Toll-like receptor 4 contributes to vascular remodelling and endothelial dysfunction in angiotensin II-induced hypertension. Br. J. Pharmacol. 172, 3159–3176 (2015). 25712370 10.1111/bph.13117 PMC4459031

[b49] Silvers A. L., Bachelor M. A. & Bowden G. T. The role of JNK and p38 MAPK activities in UVA-induced signaling pathways leading to AP-1 activation and c-Fos expression. Neoplasia (NewYork) 5, 319 (2003). 10.1016/S1476-5586(03)80025-8 PMC1502419 14511403

[b50] Ahn C. B. . Gallic Acid-Chitosan Modulates Inflammatory Responses in LPS-Stimulated RAW264.7 Cells NF-κB, AP-1 and MAPK Pathways. Inflammation 39, 366–374 (2016). 26412258 10.1007/s10753-015-0258-2

